# Miscellany

**Published:** 1896-02

**Authors:** 


### Miscellany.

MESSRS. PARKE, DAVIS & CO., the well-known manufacturing chemists, of Detroit, have issued a handsome calendar, which has for its design a pet cat evidently suffering from a big head after a night out. Its head is tied up in a bandage and one eye is swollen shut. On a table by the side of its couch are some of the medicines manufactured by this celebrated firm. "Oh! Uncle John, isn't this fun?"

---

THE Antikamnia Chemical Company, of St. Louis, has sent out to its patrons and friends a convenient pocket banknote folder, and our thanks are due to this well-known house for one of these pocketbooks. We shall try to think of antikamnia every time we file away a banknote.

